# Recent Progress of Preparation Strategies in Organic Nanoparticles for Cancer Phototherapeutics

**DOI:** 10.3390/molecules28166038

**Published:** 2023-08-13

**Authors:** Quanquan Xie, Jiayi Tang, Shengze Guo, Qi Zhao, Shengliang Li

**Affiliations:** College of Pharmaceutical Sciences, Soochow University, Suzhou 215123, China; 2130413091@stu.suda.edu.cn (Q.X.); 2130413096@stu.suda.edu.cn (J.T.); 2130413101@stu.suda.edu.cn (S.G.)

**Keywords:** phototherapy, nanoparticle, nanopreparation, co-precipitation, microfluidic, carrier-free

## Abstract

Phototherapy has the advantages of being a highly targeted, less toxic, less invasive, and repeatable treatment, compared with conventional treatment methods such as surgery, chemotherapy, and radiotherapy. The preparation strategies are significant in order to determine the physical and chemical properties of nanoparticles. However, choosing appropriate preparation strategies to meet applications is still challenging. This review summarizes the recent progress of preparation strategies in organic nanoparticles, mainly focusing on the principles, methods, and advantages of nanopreparation strategies. In addition, typical examples of cancer phototherapeutics are introduced in detail to inform the choice of appropriate preparation strategies. The relative future trend and outlook are preliminarily proposed.

## 1. Introduction

Over the past few decades, the fabrication of nanomaterials has attracted widespread interest and opened new prospects for the field of nanotechnology [1,2,3,4]. Nanoparticles (NPs) are common nanomaterials with unique performances, such as small sizes, high drug-loading capacities, and adjustable surface chemical properties, which provide them with many advantages over their volume counterparts [5]. Nanoparticles are small particles, usually smaller than 200 nanometers in diameter, which are beneficial for intracellular uptake. Nanoparticles can be used to encapsulate therapeutic drugs and release them in a controlled manner, thereby specifically targeting diseased cells [5]. The encapsulation of nanoparticles also improves the solubility of drugs [6]. In addition, other advantages of nanoparticles have also attracted widespread attention in the field of nanomedicine, including their large volume ratio, modifiable outer shell, biodegradability, and low cytotoxicity [7], bringing us closer to the comprehensive prospects of personalized medicine. The advantages of controllable delivery and modular flexibility possessed by cancer nanomedicines provide opportunities for enhancing the anti-tumor immune response and increasing the sensitivity of tumors to immunotherapy [8]. Nanoparticles can further improve the pharmacological properties of loaded immune modulators and protect biological drugs from premature release or degradation in the body. In addition, nanoparticles with adjustable physical and chemical properties (such as size, shape, and surface parameters) or multiple functions can promote inhibitory or stimulating effects on the immune system, resulting in synergistic effects on combined cancer immunotherapy [8].

The emergence of nanoparticles provides unprecedented opportunities to promote the treatment of phototherapy. We can use various nanopreparation strategies to encapsulate the phototherapy agents, thereby improving its efficacy and biocompatibility. At the same time, hidden properties of the encapsulated phototherapy agents were also activated during the nanopreparation process, including J-aggregation inducing the red-shifting of absorption and the augmented generation of reactive oxygen species (ROS) [9,10]. In addition, the high drug-loading, multimodal synergistic therapy, and integration of diagnosis and treatment can also be realized by applying appropriate preparation strategies.

**Figure 1:**
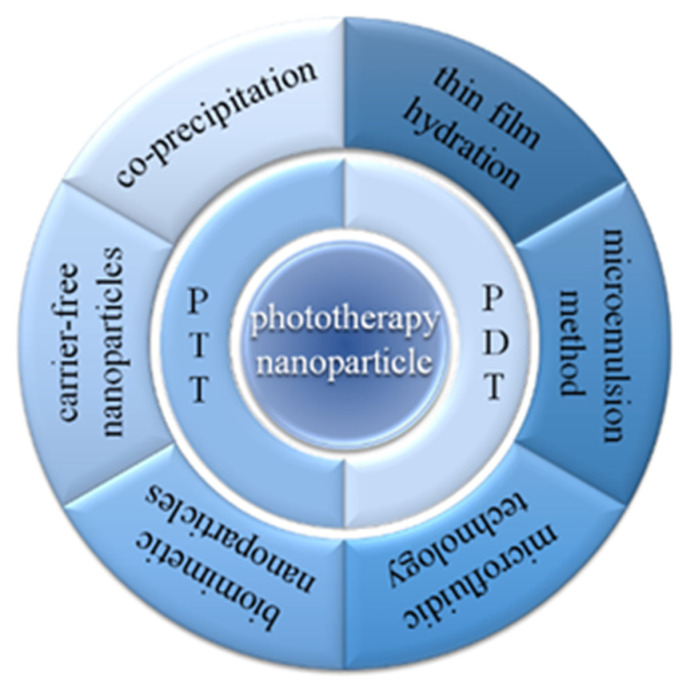
Preparation methods for phototherapy nanomedicine.

The previous reviews have introduced the preparation methods and characteristics of nanoparticles (Figure 1). This review focuses on how to choose the appropriate preparation strategies to meet phototherapy application and, recently, significant advancements in nanopreparation are also included. We expect this review will inspire researchers to develop versatile nanomaterials in the phototherapy field. 

## 2. Basic Principle of Phototherapy

**Figure 2:**
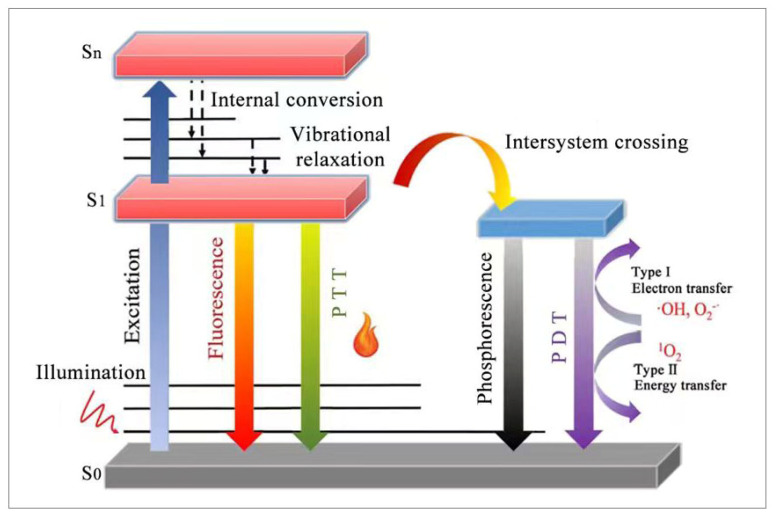
Mechanisms of phototherapy and optical imaging.

Phototherapy is considered to be an advanced treatment strategy because of its advantages of non-invasiveness, limited drug resistance, low side effects as well as high temporal and spatial selectivity [11]. Photodynamic therapy (PDT) and photothermal therapy (PTT) are the two main modalities of phototherapy (Figure 2). PDT mainly utilizes photosensitizers to boost ROS upon irradiation to perform treatments [12]. The potency of PDT is mainly achieved through three pathways: Firstly, there is the direct killing of tumor cells, leading to their necrosis. Secondly, the ROS generated by photodynamic therapy induces irreversible oxidation damage, leading to cell apoptosis. Thirdly, there is photodynamic death or the stimulation of immunogenic cell death by dead cells, resulting in a series of effects and applications in the later stage [13]. Photodynamic therapy and photothermal therapy are the two main modes of phototherapy, and they achieve the treatment of tumors by generating reactive oxygen species (ROS) and local heat; the detailed mechanism is as follows. When a suitable light is used to illuminate a photoactive molecule, the molecule’s absorbed light energy transitions from the singlet ground states (S_0_) to the singlet excited states (S_1_). Molecules in the singlet ground states are unstable and will transition back to the ground states by three relaxation modes. (1) They return to the singlet ground states by a radiation transition to produce fluorescence, which is the basic principle of fluorescence imaging. (2) Heat is generated by returning to the singlet ground states through vibration relaxation, which is the basis for photothermal therapy. (3) Intersystem crossing (ISC) occurs, producing molecules in excited triplet states, and then energy transfer (Type-II) or electron transfer (Type-I) occurs between molecules and the surrounding oxygen, generating singlet oxygen and free radicals, and is the basis for photodynamic therapy [14,15]. Although phototherapy has been proven effective in tumor treatment, its clinical application still faces many challenges. Phototherapy has great advantages over traditional cancer treatments, but its clinical application still faces challenges. First of all, limited by the tissue penetration of the laser, phototherapy can currently only treat superficial tumors. Then, poor targeting of tumor tissue may cause reduced therapeutic efficacy and increases toxic side effects. Finally, for photodynamic therapy, the high consumption on oxygen severely limit the treatment performance. For photothermal therapy, local high temperature may cause side effects such as inflammation, while low temperature may induce the expression of a heat shock protein in tumor cells and cause poor treatment effect. Therefore, the precise control of the local heat is also a major challenge for further clinical application [16]. In recent years, researchers have continuously improved it through different aspects, including the continuous exploration and optimization of the preparation method of phototherapy nanoparticles [17,18,19].

## 3. Preparation Strategies of Organic Nanoparticles

Nanoparticles can be combined with phototherapy to significantly reduce the survival rate of cancer cells. Nanoparticles can also provide optimal treatment time through imaging techniques [20]. Nanoparticles allow the simultaneous delivery of photoreactive agents and immunomodulators, so nanoparticle-based drugs are widely used in optical medicine [21]. Depending on the enhanced permeability and retention (EPR) effect, nanoparticles can be used to passively target phototherapy agents to cancerous sites [22,23,24]. In addition, the nanocarrier system can be continuously optimized to improve its permeability. Here, we introduce some of the common preparation methods including co-precipitation, thin-film hydration, microemulsion, microfluidic technology, biomimetic nanoparticles, and carrier free phototherapy, as well as their improvements.

### 3.1. Co-Precipitation

Co-precipitation is a relatively simple and inexpensive nanopreparation strategy, which is widely used in the preparation of organic nanoparticles. In fact, it is a solvent replacement method, and its basic principle is the self-assembly, nucleation and precipitation of organic molecules dissolved in it after solvent replacement [25]. Usually, the precursors (e.g., polymers, organic dyes, phototherapeutic drugs, etc.) are dissolved into organic reagents (e.g., tetrahydrofuran, methanol, dimethyl sulfoxide). Then, this organic reagent mixture is rapidly injected into the aqueous phase. After mixing, the uniformed NPs are achieved by ultrasound [26].

**Figure 3:**
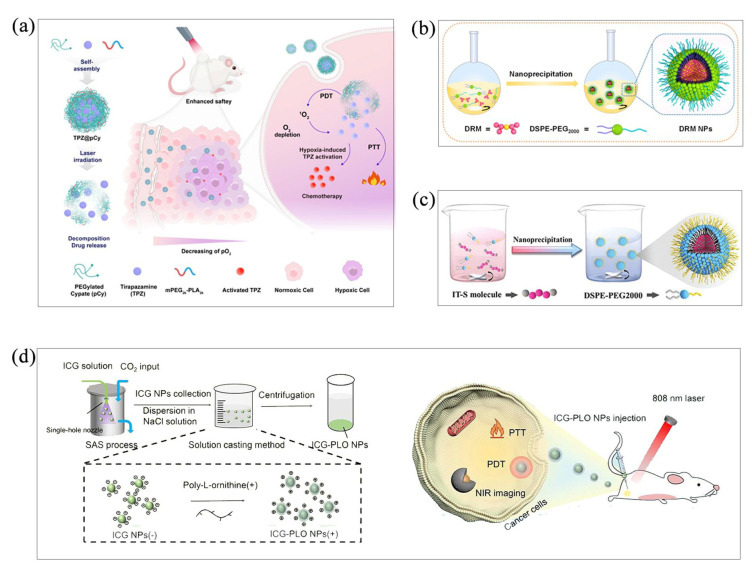
(**a**) Fabrication of TPZ@pCy and schematic representation of the application of hypoxia-enhanced phototherapy. Reproduced with permission from [27], copyright 2022 Pharmaceutics. Schematic representation of the preparation of (**b**) DRM NPs (reproduced with permission from [28], copyright 2021 ACS Appl. Mater. Interfaces) and (**c**) IT-S NPs by co-precipitation (reproduced with permission from [29], copyright 2022 ACS Small). (**d**) Schematic representation of ICG-PLO nanoparticles prepared by SAS method. Reproduced with permission from [31], copyright 2022 Int. J. Pharm.

Organic nanoparticles prepared by co-precipitation have good stability [27]. Co-precipitation has been widely used to produce organic nanoparticles for phototherapy. Here, Miao’s group explored a NIR light-decomposable nanomicelle using the re-precipitation method. The nanomicelles, consisting of pegylated cypate (pCy) and mPEG-polylactic acid, are designed for the controlled delivery of a hypoxia-activated bio-reducing prodrug (tirapazamine, TPZ), and the hypoxia-enhanced phototherapy process was conducted to combat metastatic breast cancer [27] (Figure 3a). Our group has also explored a double radical molecule (DRM) with a nano-coprecipitation method (Figure 3b). Donor-acceptor interaction forces in DRM lead to charge transfer with a distinct biradical character that favors NIR absorption. After fabrication into NPs, they achieve excellent water dispersibility, photostability and photoacoustic imaging-guided PTT performance, both in vitro and in vivo [28]. In addition to photoacoustic guidance, simple and versatile conjugated oligomeric nanoparticles (IT-S NPs), with dual imaging guidance with fluorescence and photoacoustics, have also been successfully prepared by the co-precipitation method. IT-S NPs realize precise and high-performance PTT on cancer treatments both in vitro and in vivo [29] (Figure 3c).

Although this method is simple and effective for the preparation of most organic nanoparticles, the conventional co-precipitation method is difficult to precisely control the particle morphology and particle size distribution (PSD). In addition, residual organic solvents can affect the stability of nanoparticles. With the development of new technologies, the co-precipitation method is also being optimized, among which supercritical antisolvent precipitation (SAS) has been successfully applied to the preparation of nanoparticles [30]. Chen’s group prepared ICG-PLO NPs according to the SAS method and solution casting method; this unique nanoplatform with ultra-high drug encapsulation efficiency remarkably improved the aqueous and photothermal stability of Indocyanine green (ICG). In brief, the precooled CO_2_ was continuously injected into the SAS precipitation vessel at a certain flow rate by a CO_2_ pump and heated by an electric preheater to achieve a predetermined supercritical fluid condition. After stabilization of the system, the ICG solution was injected into the system through a stainless-steel single nozzle. CO_2_ was injected continuously for 10 min to remove the solvent. After decompression, ICG particles were collected into a SAS precipitation vessel. After adding ICG NPs to the NaCl solution, poly-l-ornithine (PLO) solution was added to make the nanoparticles negatively charged under dark conditions to obtain ICG-PLO NPs. Compared with the nanoparticles prepared by the traditional co-precipitation method [31] (Figure 3d), the morphology and PSD of the nanoparticles prepared by this method can be determined. In addition, the high drug encapsulation efficiency, water solubility and photothermal stability of the nanoparticles were also demonstrated.

### 3.2. Thin-Film Hydration

The thin-film hydration method, also known as the Bangham method, is the oldest, most common and simplest method for the preparation of liposomal nanoparticles. Phospholipids were first formed into membranes, and then liposomes were formed under the action of the buffer. To ensure a homogeneous mixture, the major phospholipid component is dissolved in a round-bottom flask of a rotary evaporator with an organic reagent, such as dichloromethane, chloroform, ethanol, or a chloroform-methanol mixture, which is then sequentially evaporated under a vacuum pump at a temperature of 40–60 °C, allowing the organic reagents to be removed. The organic reagents were removed to form a uniform, dry and thin lipid film (stacked in two layers). An aqueous medium, a buffer, is then added to the membrane at a temperature higher than the transition temperature of the surfactant, and multilayer vesicles can be produced for a period of time under constant gentle stirring. The encapsulated drug was dissolved in the aqueous or organic phase depending on its solubility. This technique is usually followed by sonication to form microcapsules with uniform particle size distribution [32,33].

**Figure 4:**
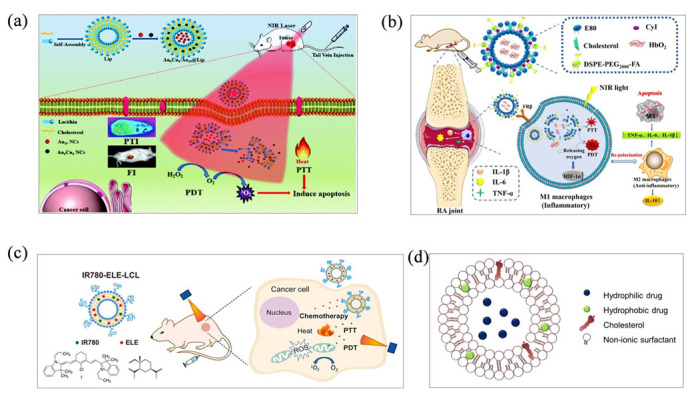
(**a**) The Au_4_Cu_4_/Au_25_ NCs were prepared by thin-film hydration to achieve dual phototherapy and dual imaging schematic. Reproduced with permission from [35], copyright 2021 J Mater Chem B. (**b**) Schematic of IR780-ELE-LCL liposomes with excellent photodynamic effects prepared by thin-film hydration. Reproduced with permission from [36], copyright 2022 J Drug Deliv Sci Technol. (**c**) Schematic illustration of the novel liposomal nanoparticles (CyI and Hb/FA-LPs) prepared by the thin-film hydration method as photosensitizers. Reproduced with permission from [37], copyright 2023 Chem. Eng. J. (**d**) Niosomes were prepared by thin-film hydration. Reproduced with permission from [32], copyright 2022 Methods.

Many lipophilic and hydrophilic drug molecules have been successfully encapsulated in liposomal nanoformulations by thin-film hydration. As carriers of phototherapy agents, liposomes have the following advantages: low toxicity and immunogenicity, and biodegradability. Both lipophilic and water-soluble phototherapy agents can be encapsulated. Liposomes can be freeze-dried and stored for a long time, which is conducive to mass production. In addition, liposomes can be prepared by a dehydration–rehydration method without using organic solvents, and the preparation method is more flexible [34]. Due to the outstanding properties of liposomes in phototherapy such as high biocompatibility, stability and targeting, thin-film hydration has been widely used to prepare phototherapeutic nanomedicine. Zhu’s group fabricated Au_4_Cu_4_/Au_25_ NCs liposomes by a simple thin-film hydration and subsequent extraction process (Figure 4a), successfully assembling a cancer treatment nanoplatform with dual imaging, dual phototherapy and laser responsiveness to the tumor microenvironment. The prepared nanoparticles have high biocompatibility, stability and passive targeting [35]. Du’s group prepared long-circulating liposomes IR780-ELE-LCL by co-loading β-element (ELE) and IR780 (Figure 4b). IR780-ELE-LCL produced a large amount of ROS under NIR irradiation and exhibited an excellent photodynamic effect. The preparation method is simple: soybean lecithin, cholesterol, DSPE-mPEG2000, ELE and IR780 are dissolved in dichloromethane, and the resulting films are spin-dried and hydrated with phosphate-buffered saline. Finally, IR780-ELE-LCL was obtained by ultrasonic and multiple membrane filter extrusion [36]. Cao’s group designed and developed novel liposomal nanoparticles (CyI&Hb/FA-LPs) by binding heavy atom-modified cyanine dye (CyI) as a photosensitizer and loading red blood cells (Hb) into the inner core to alleviate the hypoxic microenvironment and repolarize M1-to-M2 macrophages (Figure 4c). At the same time, folic acid was attached to the surface of the liposome to achieve the active targeting of M1 macrophages [37].

Even though thin-film hydration is currently one of the most common methods for liposome preparation, it is difficult to achieve mass production and a high market size. Eissa’s group mainly used thin-film hydration to prepare niosomes (Figure 4d). Niosomes are non-ion-based vesicles with great potential in the field of phototherapy due to their unique characteristics and ability to encapsulate hydrophilic and lipophilic substances [32]. Compared with the preparation of liposomes, niosomes do not require complex processes and high costs, and are easier to produce on a large scale. At the same time, niosomes have a higher stability and longer shelf life [32].

### 3.3. Microemulsion Method

The microemulsion method is an efficient method for micelle preparation. There has been an upsurge of interest on the study of microemulsions due to their unique physicochemical properties, such as thermodynamic stability, transparency and low viscosity [38,39]. Microemulsions are well-known as the transparent, isotropic micro-heterogeneous system based on a polar solvent, an amphiphilic compound and a nonpolar solvent. Moreover, the formation mechanism of microemulsion is the instantaneous negative interfacial tension mechanism [38,39]. Micelle preparation using the microemulsion method requires mixing the water and non-polar solvent, adding a surfactant and cosurfactant, stirring well, and then adding the to-be-coated drug to obtain micelle-coated nanomedicine.

**Figure 5:**
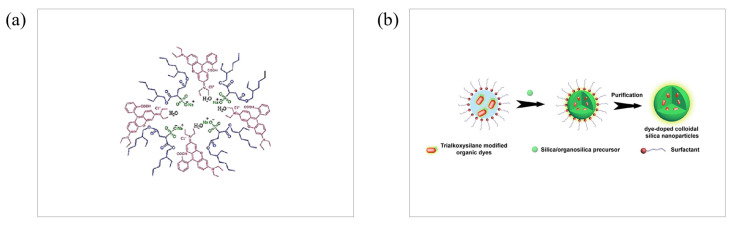
(**a**) Schematic of AOT nanodroplet containing RhB. Reproduced with permission from [40], copyright 2022 Appl. Phys. A. (**b**) Preparation process by reverse microemulsion method. Reproduced with permission from [41], copyright 2022 WIREs Nanomed Nanobiotechnol.

The nanoparticles prepared by the microemulsion method have the characteristics of narrow particle size distribution, controllable particle size and good dispersion. The microemulsion method is a simple and effective method for the preparation of organic nanoparticles, and it does not require expensive or specialized instruments. Therefore, the microemulsion method is now widely used in nanomedicine for phototherapy. Bagheri’s group prepared Rhodamine B (RhB)-containing nanodroplets in water-in-oil AOT microemulsion, and studied the photophysical properties of the dye (Figure 5a), including the excitation-ground dipole moment ratio of RhB in the nanodroplets and the stokes shift of RhB, as well as the apparent refractive index of the nanodroplet and the solvent polarity of the microemulsion [40]. In a recent report, Li’s group obtained the fluorescent colloidal silica nanoparticles by reverse microemulsion method, which ensured the ideal size control and facile surface functionalization of nanoparticles (Figure 5b). Biodistribution, targeting effects, and real-time tracking of nanomedicine in vitro or in vivo can be achieved by endografting silica nanoparticles with fluorescence properties [41]. Maake’s group attempt by the photosensitizer (PS)m—tetrahydroxyphenylchlorin (mTHPC) encapsulated into a biocompatible nanoemulsion (Lipidots) to overcome the poor water-solubility of the PS; after treatment, the patient’s skin photosensitivity was prolonged and some progress was made [42].

The advantages of nanoemulsions in PDT have attracted much attention from researchers. Although the traditional PDT technology has many advantages, the efficiency of PDT depends on the physical characteristics of PS, and the hydrophobicity of PS leads to its easy aggregation in aqueous media, poor biodistribution, and low bioavailability, which seriously limits the efficacy of PDT. Azevedo’s group developed a stable alumine-phthalocyanine chloride nanoemulsion with strong in vitro photodynamic activity against cancer cells by spontaneous emulsification. Its photophysical stability did not change significantly over 365 days [43].

### 3.4. Microfluidic Technology

Microfluidic technology has attracted much attention due to its ability to finely control the fluid flow and reaction conditions. In the past few decades, many groups have prepared nanoparticles, such as organic/inorganic semiconductors, metals and polymers, by microfluidic methods [44]. Microfluidics is a bottom-up approach that controls the fluid at the microscopic scale for the purpose of controlling the size [45,46]. Microfluidics provide fine control during automated multiroute synthesis. Fluid is manipulated within microchannels, and unlike turbulence at the macroscopic scale, fluid flow in millimeter-to-nanometer-scale channels is laminar in nature and easier to manipulate. In addition, the high specific surface area ensures a homogeneous reaction environment and efficient heat conduction, and the kinetic parameters can be accurately controlled in the state of continuous fluid [44,47].

Because of the controllability of its size, microfluidics can well meet the size requirements of phototherapy nanodrugs. Common nanomaterials such as liposomes have good thermal conductivity and have certain potential in the field of photothermal therapy.

**Figure 6:**
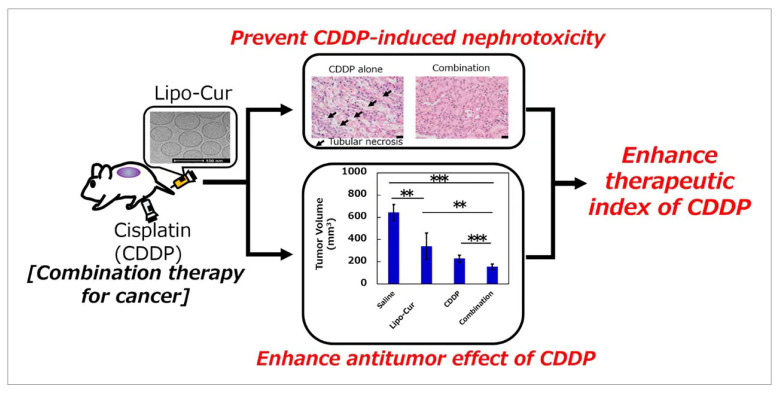
Curcumin-loaded lipo-cur was prepared by microfluidic technology to control the size of nanoparticles and improve the water solubility of the drug. ** *p* < 0.01, *** *p* < 0.005. Reproduced with permission from [50], copyright 2019 Mol. Pharm.

The ideal size of liposomes is between 50 and 200 nm, which is difficult to achieve by general methods. It is necessary to control the size of liposomes by physical methods, and microfluidics can solve this problem well. Moreover, the water solubility of the liposome nanoparticles was also improved [48,49]. Curcumin can exhibit potent anti-cancer activity through a variety of mechanisms, but its in vivo activity was affected by poor solubility. Li’s group used the microfluidic method to optimize the formulation of curcumin (lipocoagulation) liposomes (Figure 6). While precisely controlling the size of the nanomaterials, Lipo-cur simultaneously increased the water solubility of curcumin by a factor of 700, resulting in an 8- to 20-fold increase in systemic exposure compared to standard curcumin suspensions [50].

### 3.5. Biomimetic Technique

Biomimetic nanomedicine is fabricated by directly coating the cell membrane on the surface nanoparticles. This technique not only preserves the unique physiological function of nanoparticles (EPR effect), but also enables the unique physiological function of nanoparticles to be exerted. In brief, the cell membranes were collected from lysed cells and the cellular contents were removed by centrifugation. Subsequently, the nanoparticles and cell membrane were put into a centrifuge tube in a certain proportion, and PBS buffer was added to obtain the mixture. The mixture was sonicated and repeatedly extruded through the polycarbonate membrane to obtain the cell membrane-coated mimetic nanoparticles [51,52,53,54].

**Figure 7:**
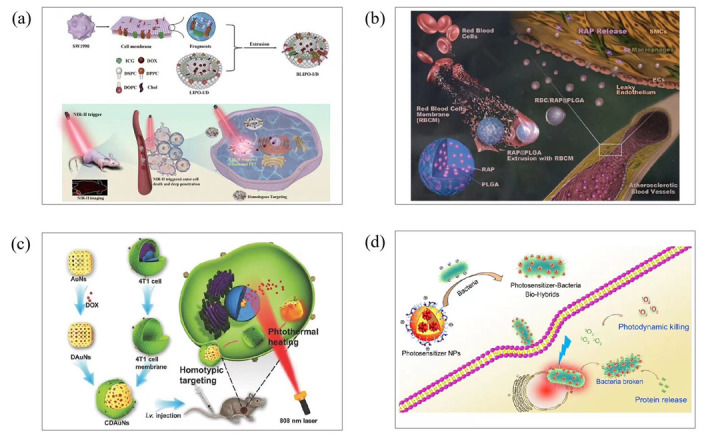
(**a**) Schematic of the preparation process of BLIPO-I/D nanoparticles and their active targeting delivery and targeted theranostics towards pancreatic tumor. Reproduced with permission from [55], copyright 2022 Mater. Today Chem. (**b**) Schematic of a 4T1 cancer cell membrane-coated gold nanocage for hyperthermia-triggered DOX release and targeted therapy of breast tumor growth and metastasis. Reproduced with permission from [56], copyright 2020 Adv. Funct. Mater. (**c**) Illustrations displaying the preparation of RBC/RAP@PLGA. Reproduced with permission from [57], copyright 2019 Adv. Sci. (**d**) Schematic of the drug delivery mechanism of bacterial vectors. Reproduced with permission from [60], copyright 2019 Chem. Mater.

Cell membrane-encapsulated nanoparticles have the characteristics of long circulation time in vivo, immune escape and homologous targeting. The biomimetic nanomedicine can enhance PTT. Therefore, biomimetic nanomedicine has a good development prospect in phototherapy. Zhang’s group created BLIPO-I/D, a biomimetic nanomedicine made by cloaking ICG-DOX liposomes with SW1990 pancreatic cancer cell membranes (Figure 7a). With the aid of homologous targeting of cell membranes, nanoparticles are enriched at the tumor site, enhancing the intensity of near-infrared fluorescence imaging. Near-infrared light (808 nm) was used to irradiate the BLIPO-I/D absorbed by pancreatic tumor tissue, triggering the rapid release of doxorubicin (DOX), and inducing the photothermal and photodynamic effects of ICG for ablation of tumors [55]. Li’s group used DOX-loaded gold nanocages (AuNs) and 4T1 cancer cell membranes (coated surface of DOX-incorporated AuNs (CDAuNs)) to set up a bionic drug delivery system (Figure 7b). This system uses cancer cell membranes for targeted drug delivery and thermotherapy, combining CDAuNs with photothermal properties to achieve the selective targeting of tumor cells and release of drugs under near-infrared laser irradiation, providing a combination of chemical and photothermal therapy [56]. In Wang’s group, the cell membrane of red blood cells was used to encapsulate rapamycin-loaded poly (lactic-co-glycolic acid) (PLGA) nanoparticles (Figure 7c). This biomimetic nanoparticle showed a clear “core-shell” structure. Therefore, it had a good hydrodynamic size and negative surface charge. In addition, the biomimetic nature of the erythrocyte membrane leads to reduced macrophage-mediated phagocytosis in the blood [57].

Although biomimetic nanofabrication methods based on the cell membrane coating have demonstrated great progress in recent years, in order to develop multifunctional and smart cell-membrane-coated nanoparticles, some modifications to the membrane are unavoidable; therefore, some side effects may occur. Excessive use of nanoparticles encapsulated in immune cell membranes may induce or aggravate inflammation through interaction with the immune system, which may lead to the release of pathological media-tors.

Therefore, many groups use bacterial carriers to produce biomimetic nanomedicine as a new method for biomimetic nanoparticle preparation. Bacterial vectors have the unique ability to preferentially colonize tumors through oxytaxis or chemotactic pathways. Their inherent genetic systems can also allow viable bacteria to be genetically engineered to deliver tumor microbicides, such as genes or proteins [58,59]. Biomimetic nanomedicine using bacteria as a carrier is usually achieved by a co-culture. Liu’s group first designed and synthesized an AIE PS (TD). The TD was then encapsulated with a biocompatible block lipid-PEG copolymer (DSPE-PEG2000) as a polymer matrix to form TD nanoparticles (TDNPs). Furthermore, a cationic polymer polyethylenimine was employed to assist in the coating of TDNPs on the surface of E. coli (TDNPP–E. coli) (Figure 7d). Bacteria-delivered multifunctional TDNPPs exhibit improved cancer cell imaging and light-mediated cancer killing in vitro compared to PS NPs without the bacteria carrier [60].

### 3.6. Carrier-Free Nanoparticle

Nanoparticles can be prepared without carriers, and are called carrier-free nanoparticles [61]. The assembly of the carrier-free nanoparticle mainly relies on non-covalent interaction such as hydrophobic interaction, π-π stacking, hydrogen bonding, and electrostatic interaction [62,63,64,65]. In brief, carrier-free nanoparticles could be prepared by injecting organic solvent including phototherapy agents dropwise into water with stirring and ultrasonication. Carrier-free nanoparticles have been proven to have the following advantages: high drug-loading capacity and good biosafety. Recently, carrier-free nanoparticles have been widely used in phototherapy, and a lot of progress has been made.

**Figure 8:**
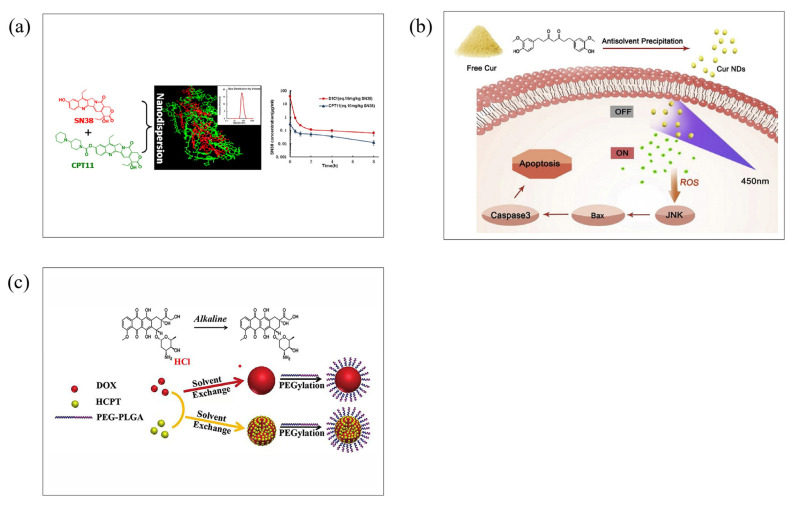
(**a**) Schematic of using CPT11 as a surfactant to induce CPT11 self-assembly with SN38 to improve drug-loading. Reproduced with permission from [66], copyright 2015 J Control Release. (**b**) Schematic representation of carrier-free nanoparticle (Cur NPs) with good water stability and exhibiting unique properties in photosensitive drug release methods. Reproduced with permission from [67], copyright 2019 Colloids Surf. B. (**c**) Preparation of carrier-free doxorubicin nanoparticles (DOX NPs). Reproduced with permission from [69], copyright 2015 Nanoscale.

For use in phototherapy, carrier-free nanoparticles have significant advantages over carrier-assistant nanoparticles in terms of preparation process, drug-loading capacity, and drug delivery efficiency, making them ideal candidates for the future clinical transformation of phototherapy [61]. Hu’s group reported that clinical amphiphilic drug irinotecan hydrochloride (CPT11) can be used as a surfactant to induce the self-assembly of CPT11 and SN38 (Figure 8a). The water solubility of SN38 was increased 1000 times after being fabricated as carrier-free nanoparticles, with a drug-loading rate close to 100%, thereby improving its bioavailability and anticancer activity [66]. Sun’s group prepared the carrier-free Curcumin (Cur) nanoparticle (Cur NDs) without using any toxic solvents (Figure 8b). The obtained Cur NDs exhibit good water stability within 7 days and can achieve drug release initiated by light. In addition, Cur NDs generate a large amount of ROS under light by photodynamic process, further activating the JNK/caspase-3 signaling pathway, inducing cell apoptosis, and making Cur NDs exhibit significantly better cytotoxicity than free Curs [67]. 

Our research group also explored carrier-free nanoparticles for effective photothermaltherapy towards cancer. Carrier-free nanoparticles, called DCF-P, are produced using the self-assembly of IDIC-4F, where a non-covalent interaction contributes to the formation. The results show that the nanocarrier DCF-P has better photothermal properties than the nanocarrier DCF-M. [68]. Doxorubicin (DOX) has been widely used in cancer therapy for its efficacy and price, and it is usually delivered in a nanocarrier. However, the low drug-loading of DOX limits its further development in most nanocarrier-based delivery systems. Zhang’s group first reported carrier-free doxorubicin nanoparticles (DOX NPs) (Figure 8c). The drug payload reaches as high as 90.47%, which greatly improves the drug payload. At the same time, it also shows good biocompatibility and stability [69].

## 4. Conclusions and Outlook

Phototherapy agents are the core of achieving efficient phototherapy for tumors. Different nanopreparation strategies will give phototherapy agents different properties to meet the needs of practical treatments. The co-precipitation method is the simplest and most widely used method. In generally, organic phototherapy agents are lipophilic molecules; thus, biological applications can be quickly realized by co-precipitation. However, for water-soluble phototherapy agents such as cyanine dyes, it is difficult to encapsulate them into nanoparticles by co-precipitation. At this time, the preparation of water-soluble phototherapy agents into liposomes is a better choice. In addition, when photothermal agents are trapped in liposomes, they can also achieve thermally responsive drug release, which is a common strategy for designing smart phototherapy materials. When there are requirements for the morphology and size of phototherapy materials, microfluidic methods can be used to control these properties, precisely. The microemulsion method can also obtain nanoparticles with narrow particle size distribution, and does not require additional equipment, and it is easier to operate than the microfluidic method. The tumor targeting of phototherapy materials is closely related to biological safety, and the use of cell membranes to coat phototherapy materials and prepare biomimetic nanoparticles to achieve homologous targeting can effectively reduce side effects and improve the efficiency of the tumor treatment. Another method to improve the efficiency of phototherapy is to prepare nanoparticles with high drug-loading by using a carrier-free nanopreparation strategy. With the aid of the carrier-free preparation strategy, the concentration of phototherapy agents at the tumor sites can be increased, and the toxicity of the carriers can be eliminated.

Nanoparticles used in phototherapy have been gradually understood, and have the advantages of prolonged circulation in vivo, special targeting and low drug toxicity; thus, it has wide prospects in phototherapy. In this review, a variety of nanoparticle preparation methods, including coprecipitation, thin-film hydration, the microemulsion method, microfluidic technology, the biomimetic technique and the carrier-free nanoparticle, as well as their principles, advantages and disadvantages, are summarized. In this review, we detail examples of phototherapy nanoparticle preparation using the above methods. The properties of nanoparticles are affected by the preparation method, and it is necessary to choose the appropriate preparation method according to the practical application. For example, nanoparticles prepared by the traditional co-precipitation method have uneven particle size distribution and poor stability. Thin-film hydration is relatively common in the laboratory, but it is not suitable for the mass production of nanomedicine. The targeting and long circulation in vivo of the biomimetic technique make it a hot research topic, but the problem of low drug-loading still needs to be solved. In addition, how to remove organic solvents has become one of the challenges in the nanopreparation process. Organic reagents are harmful to human health, which may cause inflammation, cancer and even irreversible nerve damage. Nowadays, purification techniques, including liquid extraction, membrane separation, ion exchange, electrodialysis, and reactive distillation, are used to reduce residual organic solvents with success [70,71,72,73]. We believe that improving nanopreparation strategies, such as dehydration–rehydration methods for the fabrication of liposomes, without the use of organic solvents, show great promise [74]. In view of the shortcomings of different nanoparticle preparation methods, researchers still need to further improve the corresponding methods or explore new nanoparticle preparation methods. The bioavailability, stability, cost-effectiveness, and feasibility of the preparation methods should be further developed and evolved.

## Data Availability

Not applicable.
